# The membrane topology of immunity proteins for the two‐peptide bacteriocins carnobacteriocin XY, lactococcin G, and lactococcin MN shows structural diversity

**DOI:** 10.1002/mbo3.957

**Published:** 2019-10-30

**Authors:** Angelle P. Britton, Sarah R. van der Ende, Marco J. van Belkum, Leah A. Martin‐Visscher

**Affiliations:** ^1^ Department of Chemistry The King's University Edmonton AB Canada; ^2^ Department of Chemistry University of Alberta Edmonton AB Canada; ^3^ Present address: Department of Biochemistry & Molecular Biology Dalhousie University Halifax NS Canada

**Keywords:** bacteriocin, immunity protein, lactic acid bacteria, membrane protein topology, two‐peptide

## Abstract

The two‐peptide bacteriocins produced by Gram‐positive bacteria require two different peptides, present in equimolar amounts, to elicit optimal antimicrobial activity. Producer organisms are protected from their bacteriocin by a dedicated immunity protein. The immunity proteins for two‐peptide bacteriocins contain putative transmembrane domains (TMDs) and might therefore be associated with the membrane. The immunity protein CbnZ for the two‐peptide bacteriocin carnobacteriocin XY (CbnXY) was identified by heterologously expressing the *cbnZ* gene in sensitive host strains. Using protein topology prediction methods and the dual *pho‐lac* reporter system, we mapped out the membrane topology of CbnZ, along with those of the immunity proteins LagC and LciM for the two‐peptide bacteriocins lactococcin G and lactococcin MN, respectively. Our results reveal wide structural variety between these immunity proteins that can contain as little as one TMD or as many as four TMDs.

## Graphical abstract

Through heterologous expression in a sensitive strain, we determined that CbnZ, a 42‐residue protein, confers immunity against the two‐peptide bacteriocin CbnXY. Using the dual pho‐lac reporter system, we determined the membrane topology of CbnZ, as well as the immunity proteins for lactococcin G and lactococcin MN. Our results reveal that the immunity proteins for the two‐peptide bacteriocins are a structurally diverse group of proteins.

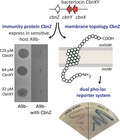

## 1 INTRODUCTION

Bacteriocins are ribosomally synthesized antimicrobial peptides produced by bacteria as a means to compete in an ecological niche. Bacteriocin production is widespread among prokaryotes (Dobson, Cotter, Ross, & Hill, 2012), and recent genome mining studies suggest that most Gram‐positive bacteria harbor genes encoding bacteriocin production (Alvarez‐Sieiro, Montalbán‐López, Mu, & Kuipers, 2016; Zhao & Kuipers, 2016). The bacteriocins produced by lactic acid bacteria have been extensively studied due to their importance within the food industry as both starter cultures and natural biopreservatives (Cleveland, Montville, Nes, & Chikindas, 2001; Juturu & Wu, 2018; Perez, Zendo, & Sonomoto, 2014), their potential use as probiotics (Cotter, Hill, & Ross, 2005; Dobson et al., 2012), and as therapeutics (Cavera, Arthur, Kashtanov, & Chikindas, 2015; Dicks et al., 2011; Lohans, & Vederas, 2012; Nes, Gabrielsen, Brede, & Diep, 2015). Over the years, numerous classification schemes for the bacteriocins from Gram‐positive organism have been proposed (van Belkum & Stiles, 2000; Cotter et al., 2005; Rea, Ross, Cotter, & Hill, 2011). In general, bacteriocins were grouped into two main families: class I consisted of lantibiotics, characterized by the presence of the modified amino acids lanthionine and/or methyllanthionine, whereas class II consisted of small, unmodified peptides. However, with the ever‐increasing discovery of bacteriocins and their structures, it has become apparent that this scheme is too restrictive. For this reason, an updated classification system has recently been proposed, in which class I broadly contains any post‐translationally modified bacteriocin, class II still consists of unmodified peptides, and class III includes large, heat labile bacteriocins such as bacteriolysins and tailocins (Acedo, Chiorean, Vederas, & van Belkum, 2018; Alvarez‐Sieiro et al., 2016).

**Figure 1:**
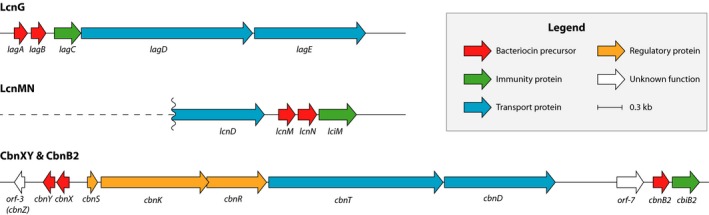
Schematic representation of the gene clusters for lactococcin G (accession number: FJ938036.1) (Nes et al., 1995) and carnobacteriocin XY (accession number: L47121.1) (Quadri et al., 1997), and of partial gene cluster for lactococcin MN (accession number: MN231270) (van Belkum et al., 1991). Open reading frames (ORFs) are indicated by arrows in the proposed direction of transcription. For ORFs with deduced functions, the color of the arrow indicates function, as listed in the legend

The linear two‐peptide bacteriocins are class II bacteriocins and require the combined action of two different peptides to elicit full antimicrobial activity. They are expressed as precursor peptides, with an N‐terminal leader sequence, and the core peptides contain characteristic GXXXG or GXXXG‐like motifs (in which a glycine has been replaced by alanine or serine (Nissen‐Meyer, Oppegård, Rogne, Haugen, & Kristiansen, 2010, 2011). Genetic evidence for a two‐peptide bacteriocin was first reported in 1991 (van Belkum, Hayema, Jeeninga, Kok, & Venema, 1991), and it was later identified as lactococcin MN (LcnMN) (van Belkum, 1994). A year later, lactococcin G (LcnG) was the first two‐peptide bacteriocin to be isolated (Nissen‐Meyer, Holo, Håvarstein, Sletten, & Nes, 1992). Since then, numerous other two‐peptide bacteriocins have been reported. The gene clusters for these bacteriocins consist of at least five genes, which encode for the precursor bacteriocins, a dedicated immunity protein, a transport protein, and an accessory protein that may be involved in bacteriocin secretion. In many cases, additional genes that encode a three‐component regulatory system are also located on, or near, the operon with the structural genes (Nissen‐Meyer et al., 2011). The gene clusters encoding for LcnG, LcnMN, and the recently discovered two‐peptide bacteriocin carnobacteriocin XY (CbnXY) (Acedo et al., 2017; Quadri et al., 1997; Tulini et al., 2014) are illustrated in Figure 1.

Mode‐of‐action studies have revealed that many of the two‐peptide bacteriocins kill target cells by creating pores in the cell membrane, resulting in leakage of ions and small molecules, and loss of the proton motive force (Nissen‐Meyer et al., 2010, 2011). Since most of these bacteriocins display narrow spectra of activity, it is likely that they require a membrane‐associated receptor. Recently, the receptors for several different two‐peptide bacteriocins have been discovered (Ekblad, Nissen‐Meyer, & Kristensen, 2017; Heeney, Yarov‐Yarovoy, & Marco, 2019; Kjos et al., 2014; Oppegård, Kjos, Veening, Nissen‐Meyer, & Kristensen, 2016). The three‐dimensional structures of LcnG (Rogne, Fimland, Nissen‐Meyer, & Kristiansen, 2008), plantaricin EF (Fimland, Rogne, Fimland, Nissen‐Meyer, & Kristiansen, 2008), plantaricin JK (Rogne, Haugen, Fimland, Nissen‐Meyer, & Kristiansen, 2009), CbnXY (Acedo et al., 2017), and plantaricin S (Ekblad & Kristiansen, 2019) have been solved. In all cases, the peptides were unstructured in aqueous environments, but assumed helical conformations when exposed to membrane‐mimicking conditions. The immunity proteins for several two‐peptide bacteriocins are known, but to date there have been no structural studies exploring these proteins and their mechanism of immunity remains a mystery. Some immunity proteins for this class of bacteriocins show homology to the Abi family of proteins, which are putative membrane‐bound metalloproteases characterized by three conserved motifs (EXXXR, FXXXH, and an invariant histidine) (Kjos, Snipen, Salehian, Nes, & Diep, 2010), and it has been suggested that these immunity proteins function by proteolytically degrading their cognate bacteriocins (Kjos et al., 2010; Lages, Mustopa, Sukmarini, & Suharsono, 2015). In other cases, such as with LagC (the immunity protein for LcnG), it is likely that the immunity protein interacts directly both with the bacteriocin and its cellular receptor (Oppegård, Emanuelsen, Thorbek, Fimland, & Nissen‐Meyer, 2010). Mutational analysis of the immunity protein for mutacin IV has identified several key residues in the C‐terminus that are essential for immunity (Hossain & Biswas, 2012). In all cases, the immunity proteins for this class of bacteriocins are predicted to contain transmembrane domains (TMDs) (Nissen‐Meyer et al., 2011), but aside from that, there appears to be very little similarity between these proteins. Elucidating the structural features of the immunity proteins for the two‐peptide bacteriocins is crucial to understanding how these proteins impart protection to producer organisms. Here, we have identified the immunity protein for CbnXY and analyzed its membrane topology. As a comparison, we have also determined the orientation of the TMDs of LagC and LciM, the immunity proteins for LcnG and LcnMN, respectively, using the dual *pho‐lac* reporter system.

## 2 METHODS AND MATERIALS

### 2.1 Bacterial strains and culture conditions

The bacterial strains used in this study are listed in Table 1. Carnobacteria cultures were grown in brain heart infusion (BHI) media (BD Difco) at 25°C, for 18–24 hr before use. *Escherichia coli* DH5α cultures were gown in Luria–Bertani (LB) media (Fisher Scientific) at 37°C, for 16–18 hr before use. *Escherichia coli* JM109 cultures were grown in LB media containing 0.2% (v/v) glucose, at 30°C, for 16 hr before use. When necessary, antibiotics were added to media at concentrations of 15 μg/ml of chloramphenicol for carnobacteria and 50 μg/ml of kanamycin for *E. coli*, respectively. Agar plates contained 1.5% (w/v) agar, while soft agar for overlays contained 0.75% (w/v) agar. All cultures were maintained in 20% glycerol and stored at −80°C.

**Table 1:** Bacterial strains and plasmids

Strain or plasmid	Relevant phenotype and property	Source or Reference
Bacteria		
*Carnobacterium maltaromaticum* LV17B	CbnXY^+^ Imm^+^	Ahn & Stiles, 1990
*C. maltaromaticum* A9b‐	CbnXY^−^, Imm^−^	Tulini et al., 2014
*Carnobacterium divergens* LV13	CbnXY^−^, Imm^−^	Ahn & Stiles, 1990
*Escherichia coli* DH5α	Cloning host	Invitrogen
*E. coli* JM109	Expression host	Promega
Plasmids		
pMG36c	Lactococcal expression vector, Cm^r^	van de Guchte et al., 1989
pMG36c‐orf3	pMG36c containing *orf‐*3	This study
pMG36c‐orf7	pMG36c containing *orf*‐7	This study
pKTop	PhoA/LacZ dual reporter vector (PhoA_22‐472_/LacZ_4‐60_), Kan^r^	Karimova & Ladant, 2017
pKTop‐CbnZ‐41	pKTop derivative expressing CbnZ_1‐L41_/PhoA/LacZ	This study
pKTop‐LagC‐31	pKTop derivative expressing LagC_1‐K31_/PhoA/LacZ	This study
pKTop‐LagC‐56	pKTop derivative expressing LagC_1‐A56_/PhoA/LacZ	This study
pKTop‐LagC‐88	pKTop derivative expressing LagC_1‐A88_/PhoA/LacZ	This study
pKTop‐LagC‐110	pKTop derivative expressing LagC_1‐R110_/PhoA/LacZ	This study
pKTop‐LciM‐30	pKTop derivative expressing LciM_1‐K30_/PhoA/LacZ	This study
pKTop‐LciM‐60	pKTop derivative expressing LciM_1‐A60_/PhoA/LacZ	This study
pKTop‐LciM‐93	pKTop derivative expressing LciM_1‐K93_/PhoA/LacZ	This study
pKTop‐LciM‐130	pKTop derivative expressing LciM_1‐G130_/PhoA/LacZ	This study
pKTop‐LciM‐154	pKTop derivative expressing LciM_1‐K154_/PhoA/LacZ	This study

### 2.2 Construction of plasmids to identify the immunity protein for CbnXY

The plasmids and primers used in this study are listed in Tables 1 and 2, respectively. Plasmid DNA was purified using a QIAprep Spin Miniprep Kit (Qiagen). Platinum SuperFi DNA Polymerase, restriction enzymes, and T4 DNA ligase were obtained from Invitrogen and used according to the manufacturer's instructions. Using plasmid DNA from *Carnobacterium maltaromaticum* LV17B as the template, primers LMV62 and LMV63 were used to amplify *orf*‐3, and primers LMV69 and LMV70 were used to amplify *orf*‐7. The forward primers (LMV63 and LMV70) also contained the ribosomal binding site for each gene. The PCR products and constitutive expression vector pMG36c (van de Guchte, van der Vossen, Kok, & Venema, 1989) were doubly digested with appropriate restriction enzymes, ligated with T4 DNA ligase, and transformed into *E. coli* DH5α via heat shock. Transformants were selected on LB plates using chloramphenicol. Colony PCR was used to identify transformants containing *orf‐*3 or *orf*‐7, after which nucleotide sequencing of the recombinant plasmids confirmed the cloning of the genes. In the case of the plasmid containing *orf*‐3, sequencing revealed that there was insertion at the *Pst*I restriction site of ~800 nucleotides of *E. coli* DNA upstream of the *orf‐*3 gene. Thus, the plasmid was digested with *Pst*I to remove the interfering DNA and self‐ligated to yield the correct plasmid. The recombinant plasmids pMG36c‐orf3 and pMG36c‐orf7, along with pMG36c as a negative control, were then transformed by electroporation into the CbnXY‐sensitive organisms *Carnobacterium divergens* LV13 and *C. maltaromaticum* A9b‐ as described previously (van Belkum & Stiles, 2006). Transformants were grown on BHI agar plates containing chloramphenicol, at room temperature.

**Table 2:** Oligonucleotides used in this study

Primer	Sequence (5'→3')^a^	Purpose
LMV62	ATATAAGCTTTTAAAATAGGCTCCCGATAGACCT	Amplification of *orf*‐3, reverse
LMV63	ATATCTGCAGGACTAAATTAGGAGGGTTTTTATGAA	Amplification of *orf*‐3, forward
LMV69	CTTCTGCAGCTATATAACTTTTTTACGATTA	Amplification of *orf*‐7, reverse
LMV70	CTTGAGCTCTGGAGCATAAAATGAAAAAT	Amplification of *orf*‐7, forward

a Restriction enzyme cleavage sites [*Hin*dIII (AAGCTT), *Pst*I (CTGCAG), and *Sac*I (GAGCTC)] are underlined.

### 2.3 Bacteriocin activity and immunity assays

Spot‐on‐lawn testing was performed to assess the sensitivity of the transformants, the producer organism (LV17B), and the sensitive strains (LV13 and A9b‐) to purified CbnXY, as previously described (Acedo et al., 2017). Briefly, a 500 μM solution of CbnXY (total bacteriocin concentration, equimolar) was serially diluted twofold and 10 μl of each dilution was spotted onto a BHI agar plate that had been overlaid with soft agar containing the organism to be tested. Plates were incubated for ~18 hr at room temperature and inspected for zones of inhibition.

### 2.4 In silico prediction of membrane topology of CbnZ, LagC, and LciM

**Figure 2:**
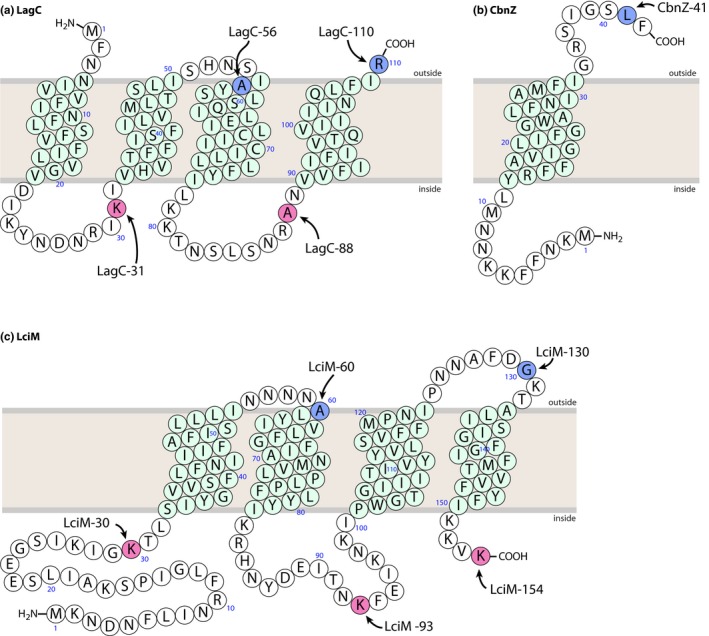
Transmembrane topology models for LagC, CbnZ, and LciM as predicted by TMHMM (http://www.cbs.dtu.dk/services/TMHMM-2.0/). Green circles represent putative helical membrane spanning domains. Fusion points with the PhoA‐LacZ reporter protein are highlighted in pink or blue and labeled. Pink and blue indicate the expected color phenotypes of cells expressing the fusion protein when grown on media containing the chromogenic substrates X‐Pho and Salmon‐Gal

Membrane topology models of CbnZ, LagC, and LciM were constructed by combining the topologies predicted by TMHMM (http://www.cbs.dtu.dk/services/TMHMM-2.0/), TMpred (https://embnet.vital-it.ch/software/TMPRED_form.html), and Protter (http://wlab.ethz.ch/protter/start/; Omasits, Ahrens, Müller, & Wollscheid, 2013). Protein sequences for CbnZ, LagC, and LciM were obtained from the NCBI protein database (https://www.ncbi.nlm.nih.gov/protein/). Using these topology models (Figure 2), proper deletion mutants were designed for each immunity protein to use with the dual *pho‐lac* reporter system.

### 2.5 Construction of fusion proteins with the dual *pho‐lac* reporter system

Truncated forms of CbnZ, LagC, and LciM were fused onto the dual PhoA‐LacZ reporter protein using the pKTop vector. The DNA encoding the truncated immunity proteins was obtained from BioBasic (Markham, Ontario) and cloned into the *Hin*dIII and *Bam*HI restriction sites of the pKTop vector. The plasmids, along with the pKTop vector as control, were transformed into *E. coli* JM109 (Promega). Transformants were grown at 30°C on LB plates containing kanamycin and glucose. Plasmid DNA from each set of transformants was isolated and sequenced to confirm that the genes were correct and in frame with the *phoA‐lacZ* reporter gene. The activity of the reporter protein for each of these transformants was examined by streaking transformants onto dual indicator LB agar plates, containing 100 μg/ml 5‐bromo‐4‐chloro‐3‐indolyl phosphate disodium salt (X‐Pho; Sigma), 80 μg/ml 6‐chloro‐3‐indoxyl‐β‐D‐galactopyranoside (Salmon‐Gal; GoldBio), 0.1 mM IPTG, and kanamycin, and incubating at 30°C for 16 hr.

## 3 RESULTS AND DISCUSSION

### 3.1 CbnZ functions as the immunity protein for CbnXY

In order to identify the immunity protein for CbnXY, we first examined the operon containing the *cbnXY* structural genes to identify which gene might encode for the immunity protein. This gene cluster (Figure 1) contains several additional genes responsible for the production of the bacteriocin CbnB2, including genes that encode for regulatory and transport proteins (Kleerebezem, Kuipers, de Vos, Stiles, & Quadri, 2001; Quadri et al., 1997). Two additional open reading frames of unknown function, *orf*‐3 and *orf*‐7, also reside in the gene cluster. Since *orf*‐3 was located immediately downstream of *cbnXY*, it was a likely candidate to encode the immunity protein. However, the gene product is a short protein (42 amino acids) with just one putative TMD. On the other hand, *orf*‐7 was located further away from *cbnXY* and on the opposite strand, but it encoded for a longer protein (109 amino acids) with three putative TMDs. As such, we decided to express both of these genes in two different CbnXY‐sensitive strains by cloning *orf‐*3 and *orf‐*7 downstream of the strong constitutive promoter of the expression vector pMG36c, giving plasmids pMG36c‐orf3 and pMG36c‐orf7, respectively, and use these plasmids to transform strains LV13 and A9b‐.

Spot‐on‐lawn testing revealed that transformants harboring either pMG36c or pMG36c‐orf7 showed no increase in resistance to CbnXY when compared to the sensitive strains, indicating that *orf*‐7 does not seem to provide protection against the bacteriocin. However, transformants with pMG36c‐orf3 displayed a 250‐fold increase in resistance to CbnXY, thus revealing that *orf*‐3 encodes an immunity protein, which we named CbnZ. Interestingly, these transformants were still susceptible to CbnXY at the highest concentrations tested, whereas the producer organism (LV17B) was completely immune. Similar observations have been reported for the heterologous expression of other immunity proteins, and it has been suggested that this difference in immunity may be due to poor levels of transcription of the immunity protein genes when under control of a different promoter in the heterologous host (Flynn et al., 2002; Quadri et al., 1995).

### 3.2 Immunity proteins for the two‐peptide bacteriocins are structurally diverse

CbnZ is a small hydrophobic protein of just 42 amino acids, and while there are a few examples of short immunity proteins for this class of bacteriocins, such as BrcI (53 amino acids), EnkIaz (52 amino acids), and AbpIM (55 amino acids) (Flynn et al., 2002; Ishibashi et al., 2014; McCormick et al., 1998), most other immunity proteins for this class of bacteriocins are substantially longer and are predicted to have at least four or five TMDs (Nissen‐Meyer et al., 2011). For example, the immunity proteins LagC (Nes, Håvarstein, & Holo, 1995) and LciM (van Belkum et al., 1991) consist of 110 and 154 amino acids, respectively, and are predicted to have at least four TMDs (Nissen‐Meyer et al., 2011). We were therefore interested in further exploring the structural diversity among these various immunity proteins by investigating whether CbnZ, LagC, and LciM are indeed membrane‐associated, and by mapping out of the orientation of the TMDs of these proteins across the membrane.

The dual *pho‐lac* reporter system of the pKTop expression vector is a convenient method to characterize the membrane topology of proteins (Karimova & Ladant, 2017). This system employs two *E. coli* proteins, alkaline phosphatase (PhoA) and β‐galactosidase (LacZ), which are only active in the periplasm and cytoplasm, respectively. PhoA converts the substrate X‐Pho into an insoluble blue compound, whereas LacZ converts Salmon‐Gal into a red precipitate. Thus, when these proteins are fused to the C‐terminus of a putative TMD, the orientation of the TMD can easily be determined. To use this system, we began by identifying the putative TMDs and loop regions for CbnZ, LagC, and LciM. Based on the results of membrane protein topology prediction methods (Figure 2), one TMD was predicted for CbnZ, and four TMDs were predicted for both LagC and LciM. These models also predicted that the N‐ and C‐termini for LagC were located on the outside of the cytoplasmic membrane, whereas the N‐ and C‐termini for LciM were predicted to be located inside the cytoplasm. From these models, we identified key residues to fuse to the *pho‐lac* system of the pKTop vector. In total, we designed and constructed ten different fusion proteins: four for LagC, one for CbnZ, and five for LciM. We used the *Hin*dIII and *Bam*HI restriction sites of the pKTop vector, which necessarily introduced an additional eight non‐native amino acids to the N‐termini of each fusion protein.

**Figure 3:**
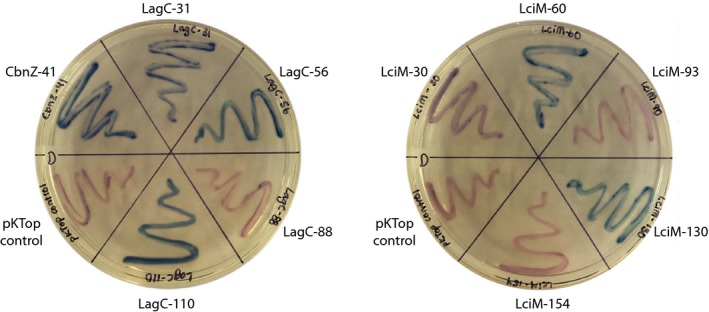
Experimental determination of the membrane topologies of LagC, CbnZ, and LciM by monitoring the activity of the PhoA‐LacZ reporter protein. *Escherichia coli* JM109 harboring the various pKTop constructs were grown on dual indicator plates contain 100 μg/ml X‐Pho and 80 μg/ml Salmon‐Gal. A red phenotype indicates high β‐galactosidase (LacZ) activity, revealing that the reporter protein has been positioned in the cytoplasm. A blue phenotype indicates high alkaline phosphatase (PhoA) activity and reveals that the reporter protein has been positioned in the periplasm. *Escherichia coli* JM109 containing the pKTop vector without insert appears pink

We determined the activity of the reporter system by growing *E.* *coli* JM109 transformants containing the various constructs on media supplemented with the appropriate chromogenic substrates. Our experimental results (Figure 3) closely matched the model predictions. CbnZ contains one TMD, with its N‐terminus in the cytoplasm and its C‐terminus on the outside of the cytoplasmic membrane. Likewise, our results confirmed that LciM has four TMDs. Fusion points LciM‐30, LciM‐93, and LciM‐154 are all located in the cytoplasm, revealing that both the N‐ and C‐termini of LciM are intracellular, and there is one intracellular loop. Fusion points LciM‐60 and LciM‐130 were located on the outside, confirming that LciM has two extracellular loops.

In the case of LagC, the fusion points of LagC‐56 and LagC‐110 were periplasmic and the fusion point of LagC‐88 was in the cytoplasm, as was expected. However, the experimental result obtained with LagC‐31 did not agree with the model prediction. We expected the N‐terminus of LagC‐31 to be positioned outside the cytoplasmic membrane and the fusion point of LagC‐31 inside the cell. This would place the reporter protein in the cytoplasm, and therefore, we expected the cells to appear red. However, *E. coli* transformants containing this construct consistently appeared blue when grown on dual indicator media, suggesting the opposite orientation across the membrane. Since this was our shortest construct, we wondered if the additional eight non‐native amino acids at the N‐terminus were influencing the protein structure and causing a reversal of orientation of the first TMD. To test this, we designed a new construct that included a stop codon immediately after the codons for the eight non‐native amino acids, followed by a ribosomal binding site and a start codon for LagC‐31. However, the resulting transformants still appeared blue when grown on dual indicator plates, indicating that the eight non‐native amino acids at the N‐terminus were not responsible for the unexpected result of LagC‐31 (data not shown).

The orientation of TMDs across a membrane is strongly influenced by the “positive‐inside rule,” and in general, topology is determined by the most positive loop (von Heijne, 1989, 2006). In the complete LagC protein, loop 1 (residues 22–32) is predicted to be inside the cytoplasm; however, this loop is only marginally positive with a pI of ~8.5, whereas the second intracellular loop between residues 78 and 89 has a pI of ~11.2 and likely dictates overall topology of the protein. In the LagC‐31 construct, incorporation of the reporter protein increases the prevalence of negatively charge amino acids, which decreases the overall positive charge of the loop 1 flanking region. Recently, it has also been shown that negatively charged residues such as aspartic acid and glutamic acid are preferentially located on the outside of the membrane, or at least suppressed on the inside (Baker, Wong, Eisenhaber, Warwicker, & Eisenhaber, 2017). The presence of aspartic acid residues at positions 22 and 27 in LagC will likely further force the TMD of the LagC‐31 construct to reverse its orientation across the membrane. This would explain the unexpected blue color of transformants containing LagC‐31. Based on our experimental data, in combination with our predicted topology models, we conclude that LagC is membrane‐associated with four TMDs that are most likely oriented as shown in Figure 2.

## 4 CONCLUSIONS

We have determined that CbnZ functions as the immunity protein for the two‐peptide bacteriocin CbnXY. To date, this is the shortest known immunity protein for this class of bacteriocins. Using the *pho‐lac* reporter system, we have also confirmed that CbnZ, along with the immunity proteins for LcnG and LcnMN, is membrane‐associated. Our results reveal that the immunity proteins for these two‐peptide bacteriocins display great structural variety: They range in length, number of TMDs, and orientation across the membrane. Our findings are an important first step toward uncovering the structural features of this intriguing group of proteins. Further investigations, such as labeling studies, may help uncover how these immunity proteins provide protection to their producer organisms.

## CONFLICT OF INTERESTS

None declared.

## AUTHOR CONTRIBUTIONS

APB, SRvdE, and LAMV performed the experiments. All authors were in involved in data analysis. LAMV and MJvB designed and supervised the study, and wrote the manuscript. All authors read and approved the final manuscript.

## ETHICS STATEMENT

None required.

## Data Availability

All data generated or analyzed during this study are included in this published article.
